# Knowledge, Attitude, and Practice Regarding the Use of Isotretinoin in Saudi Arabia

**DOI:** 10.7759/cureus.50516

**Published:** 2023-12-14

**Authors:** Walid A Alghamdi, Hussain S Alwesaibie, Mohammed A Albesher, Fai K Alghamdi, Alreem A Albaqshi

**Affiliations:** 1 Dermatology, Security Forces Hospital, Riyadh, SAU; 2 Medical School, King Faisal University, Al-Ahsa, SAU

**Keywords:** roaccutane, prevalence, knowledge, acne, isotretinoin

## Abstract

Background

Acne vulgaris is a widespread chronic inflammatory dermatological disease with a worldwide prevalence of 9.4%, affecting a large percentage of the young population in the Kingdom of Saudi Arabia. This study aimed to measure the knowledge, attitude, and practice toward the use of isotretinoin among the Saudi population.

Methodology

This cross-sectional study was conducted among the general population in Saudi Arabia between April 2023 and July 2023. A self-administered questionnaire was distributed across five regions of Saudi Arabia (central, eastern, western, southern, and northern) using Google Survey. The questionnaire included sociodemographic data, the pattern of isotretinoin used, and a 24-item questionnaire to assess the knowledge about isotretinoin.

Results

Of the 3,001 participants, 77.1% were females, and 55.4% were aged between 16 and 25 years. The prevalence of participants who previously used isotretinoin was 30.6%. The total mean knowledge score was 10.4 (SD = 5.99) out of 24 points, with more than half (52.9%) considered to have poor knowledge, 39.1% moderate knowledge, and only 7.9% good knowledge. Younger participants, female gender, Saudi nationality, never been married, and previous use of isotretinoin were associated with increased knowledge.

Conclusions

The knowledge of the general population regarding isotretinoin use was lacking. However, younger Saudi females who had previous isotretinoin usage tended to be more knowledgeable about isotretinoin compared to the rest of the participants. Increasing the general public’s knowledge regarding isotretinoin and its safe use is necessary.

## Introduction

Acne vulgaris (AV) is a widespread chronic inflammatory dermatological disease with a global prevalence of 9.4%, affecting a large percentage of the young population in the Kingdom of Saudi Arabia (KSA) [1,2]. The underlying pathophysiology of AV involves the pilosebaceous unit with three major elements, namely, hyperseborrhoea, abnormal keratinization, and *Propionibacterium acnes* proliferation, which interact to change the cutaneous microenvironment, leading to inflammatory reactions of the host and promoting acne lesion evolution [3,4]. Genetic and environmental factors play important roles in its pathogenesis as well [5]. The resultant lesions range from mild physiologic acne, non-inflammatory comedones, inflammatory papules, nodules, and pustules to nodulocystic acne, scarring, and abscess formation [6,7]. AV is widely recognized for its negative impact on both physical and psychosocial health and well-being [8]. The treatment of AV ranges from topical therapy (retinoids, benzoyl peroxide, and antibiotics), oral antibiotics, and combined therapy to oral isotretinoin [9,10].

Systemic isotretinoin (13-cis-retinoic acid) is a synthetic retinoid agent (vitamin A derivative) and a licensed treatment by the United States Food and Drug Administration (FDA) since 1982 to treat severe forms of acne such as nodulocystic and conglobate acne, in case a risk of irreversible scarring is likely, or when treatment with prior topical therapy and systemic antibiotics have not been satisfying [11,12]. Isotretinoin represents the single most unrivaled advancement in acne treatment [13]. Isotretinoin tackles all acne-related processes, inhibiting sebaceous gland activity and keratinization, reducing sebum production, and causing comedolytic. As a result, the proliferation of *P. acnes* diminishes, and chemotactic inflammatory modulator release reduces, limiting the cutaneous inflammation [14,15]. AV patients who are candidates to be prescribed isotretinoin are often given a dose of 0.5-1 mg/kg/day with a fatty meal as it escalates gastrointestinal absorption. A lower dosage is given in the first month of the treatment to avert an initial acne flare and gradually aid in adapting to dose-dependent side effects. It has been established that attaining a cumulative dosage of 120-150 mg/kg decreases the likelihood of relapse. Moreover, a six-month treatment with low-dose isotretinoin may aid in treating moderate acne with fewer adverse effects and better satisfying outcomes for patients [16].

Despite its spectacular efficacy in treating AV, isotretinoin carries various adverse effects that sometimes limit its use. These side effects include dry lips, as the most commonly reported adverse event, xerosis, dry eyes, dry nose, photosensitivity, conjunctivitis, elevated serum cholesterol levels, hypertriglyceridemia, pancreatitis, elevated liver enzymes, blood dyscrasias, hyperostosis, psychiatric disorders such as depression (though these are extremely rare), and teratogenicity, a major adverse event [17,18]. Therefore, isotretinoin should be prescribed by physicians with adequate experience in its use and enough knowledge about its risks and monitoring requirements [19].

Furthermore, AV can have a negative impact on a patient’s health-related quality of life (HRQoL) that further worsens based on the acne’s severity, along with the possibility of developing psychological impairment in the form of anxiety, low mood, and suicidality [20]. Eventually, given AV prevalence is continuously rising along with the prescription of isotretinoin and the associated adverse effects and negative impact on patients’ HRQoL, there is still a lack of studies assessing the knowledge, attitude, and practice (KAP) toward the use of isotretinoin targeting acne patients from the different regions of Saudi Arabia. Therefore, this study aimed to address this issue and determine the KAP toward the use of isotretinoin, its safety profile, and side effects among the Saudi population.

## Materials and methods

Study methodology

This cross-sectional study targeted the general population in Saudi Arabia to determine the knowledge, attitude, and compliance about the use of isotretinoin. The study was conducted between February 2023 and May 2023. Using Google Forms, an online survey was created for data collection. People were invited to participate in the online survey, which was distributed to the general public in each of the five regions of Saudi Arabia (central, eastern, northern, western, and southern). To reach the population from each region of the five regions, and to enroll as many participants as possible, two or more data collectors were recruited from every region. Data were gathered via an online, self-administered questionnaire that participants completed after providing their consent to participate in the study. The questionnaire included three sections. The first section asked about sociodemographic data, including age, gender, nationality, residency, region, education level, occupation, marital status, acne type, daily dose of isotretinoin, and current isotretinoin course period. The second part asked about patients’ knowledge of different isotretinoin adverse effects, including skin dryness, teratogenicity, dyslipidemia, itchiness, rash, nosebleeds, joint and back pain, dizziness, eye inflammation, depression, and increased liver enzymes. The third section asked nine yes/no questions that evaluated patients’ knowledge about isotretinoin, including the necessity of a valid prescription, the length of time that isotretinoin should be used, the importance of using sunscreen when taking isotretinoin, the requirement to make regular laboratory check-ups, and the use of isotretinoin during pregnancy, lactation, and blood donation, among others. Every right response received a score of one, and the results were summed up to nine. The survey was developed by the investigators with the help of various similar studies done previously. The survey was presented to specialists in dermatology for improvement and approval which was first developed in English and then translated into Arabic for it to be comprehensible to the targeted population. The Arabic version was examined by three different language experts and the translation was approved after grammatical and linguistic modifications. Subsequently, a pilot study was performed on a small group of people (15 persons) after obtaining their consent to confirm a uniform understanding of the questions.

Study population

The study participants were the general population of Saudi Arabia who consented to participate in this between February and May 2023 and met the inclusion criteria. The inclusion criteria consisted of individuals aged 13 years and older, living in Saudi Arabia, and consenting to participate in the study. The exclusion criteria consisted of being younger than 13 years, living outside of Saudi Arabia, and not consenting to participate in the study. The sampling technique utilized was convenient random sampling where the questionnaire was disseminated via social media platforms, and the general population of Saudi Arabia was invited to participate through an online link. The following formula was used to determine the sample size: n = Z^2pq/d^2, where n is the desired sample size, Z is the statistic corresponding to the level of confidence, P is expected prevalence, q is 1-p representing the proportion in the target population not having the particular characteristics, and d is the precision (corresponding to effect size). With a 95% confidence level, 50% estimated proportion, and 5% precision level, a minimum of 385 samples was determined. However, more candidates and participants were added to ensure that the results were sufficient and accurate.

Study procedure

According to the inclusion and exclusion criteria, certain participants who fulfilled the criteria and provided consent were enrolled. Each individual anonymously filled out the questionnaire. The results of the questionnaires were analyzed statistically using the SPSS version 26 (IBM Corp., Armonk, NY, USA). Interpretation of the collected data was done accordingly, and proposals of potential solutions, when applicable, were delivered.

Data management

The questionnaire results were stored in a Google database, and only approved personnel had access to the data. Privacy and confidentiality were a priority, and no points mentioned as part of the questionnaire threatened participants’ confidentiality.

Questionnaire criteria

The knowledge about isotretinoin was assessed using a 24-item questionnaire, with yes coded with 1 and no/I don’t know coded with 0. Negative questions were coded reversely (no coded as 1; yes/I don’t know coded as 0) to avoid bias in the score. The overall knowledge score was calculated by adding all 24 items. A possible score range from 0 to 24 points was generated. The greater the score, the greater the knowledge about isotretinoin. Using 50% and 75% to determine the level of knowledge, participants were classified as having poor knowledge if the total score was less than 50%, 50% to 75% as moderate knowledge, and above 75% as having good knowledge levels.

Statistical analysis

Numbers and percentages (%) were used to represent categorical data. Continuous data were calculated and displayed as mean and standard deviation. The differences in the knowledge score regarding the sociodemographic characteristics were assessed using the Mann-Whitney Z test and the Kruskal-Wallis H test. The normality test (i.e., statistical collinearity) was performed using the Shapiro-Wilk and Kolmogorov-Smirnov tests. Based on the overall data distribution, the knowledge score followed the non-normal distribution. Therefore, the non-parametric tests were applied. Statistical significance was established at p-values <0.05. All statistical data were carefully analyzed using SPSS version 26 (IBM Corp., Armonk, NY, USA).

## Results

In total, 3,001 participants filled out the survey. As seen in Table 1, 55.4% were aged between 16 and 25 years, with females being dominant (77.1%). Nearly all (92.5%) were Saudis, and 36.6% resided in the eastern region. Single participants constituted 61.2%, and students accounted for 50.9%. The prevalence of participants who previously used isotretinoin was 30.6%.

**Table 1:** Participants sociodemographic characteristics (n = 3,001).

Study data	N (%)
Age group	
16–25 years	1,662 (55.4%)
26–35 years	620 (20.7%)
36–45 years	394 (13.1%)
>45 years	325 (10.8%)
Gender	
Male	687 (22.9%)
Female	2,314 (77.1%)
Nationality	
Saudi	2,777 (92.5%)
Non-Saudi	224 (07.5%)
Region	
Central region	708 (23.6%)
Eastern region	1,099 (36.6%)
Northern region	298 (09.9%)
Western region	644 (21.5%)
Southern region	252 (08.4%)
Marital status	
Single	1,837 (61.2%)
Married	1,057 (35.2%)
Divorced or widowed	107 (03.6%)
Occupational status	
Unemployed	1,104 (36.8%)
Student	1,527 (50.9%)
Employed in a medical field	237 (07.9%)
Employed in a non-medical field	133 (04.4%)
Have you ever used isotretinoin in the past?	
Yes	918 (30.6%)
No	2,083 (69.4%)

In Table 2, 97.5% of isotretinoin users used it to treat acne, with mild acne (39%) being the most common. Overall, 42.4% used 10-20 mg daily doses of isotretinoin. Most were prescribed by a dermatologist (89%) and used for six months or less (63%). Approximately 85.4% believed finishing the Isotretinoin course is necessary to get maximum benefit.

**Table 2:** Characteristics of participants who used isotretinoin (n = 918).

Variables	N (%)
Do you use isotretinoin as a treatment for acne?	
Yes	895 (97.5%)
No (for the treatment of other disease)	23 (2.5%)
Acne type	
Mild: less than 30 blackheads and whiteheads	358 (39.0%)
Moderate: almost 100 blackheads and whiteheads	250 (27.2%)
Severe: nodules: (large painful red lumps)	192 (20.9%)
More severe: pigmented maculed (dark marks from old spots) or scars	118 (12.9%)
Daily dosage for isotretinoin (Roaccutane)	
<10 mg	191 (20.8%)
10–20 mg	389 (42.4%)
20–40 mg	271 (29.5%)
40–60 mg	61 (6.6%)
>60 mg	06 (0.70%)
Isotretinoin (Roaccutane) has been prescribed by	
Dermatologist	817 (89.0%)
Another doctor	76 (8.3%)
Self-prescription	25 (2.7%)
How long is your current isotretinoin (Roaccutane) use?	
≤6 months	578 (63.0%)
>6 months	340 (37.0%)
It is mandatory to finish the isotretinoin course for the maximum benefit and to prevent acne from returning	
Yes	784 (85.4%)
No	38 (4.1%)
I don’t know	96 (10.5%)

Regarding the different isotretinoin side effects (Table 3), only three statements had good ratings based on the correct answers. These statements were “Isotretinoin can make your skin dry” (75%), followed by “Isotretinoin may lead to teratogenicity (fetal defects) if it takes during” (62%) and “Increase in isotretinoin dose, the more side effects you experience” (58.7%), while poor ratings were seen with the rest of the knowledge items about isotretinoin side effects, with more emphasizing on “Isotretinoin may lead to teratogenicity (fetal defects) if it takes by a male” (19.7%) and “Isotretinoin can cause many long-term adverse effects” (9.8%). Regarding the patients’ knowledge item, the top three statements where participants showed better knowledge were “Isotretinoin can be used without prescription” (75.9%), “Isotretinoin should be given with plenty of water to avoid dryness” (72.1%), and “No need to have laboratory monitoring during isotretinoin use” (63.7%). Based on the above statements, the overall mean knowledge score was 10.4 (SD = 52.9), with poor, moderate, and good knowledge levels constituting 52.9%, 39.1%, and 7.9%, respectively.

**Table 3:** Assessment of the knowledge about isotretinoin (n = 3,001).

Statement	N (%)
Knowledge about different isotretinoin adverse effects	
Isotretinoin can make your skin dry (yes)	2,250 (75.0%)
Isotretinoin may lead to teratogenicity (fetal defects) if it is taken during pregnancy (yes)	1,860 (62.0%)
Increase in isotretinoin dose, the more side effects you experience (yes)	1,761 (58.7%)
You may get a skin rash and itching during isotretinoin therapy (yes)	1,488 (49.6%)
One of the most side effects of isotretinoin is an elevation of liver enzymes (yes)	1,394 (46.5%)
Isotretinoin can lead to inflammation of the eye (yes)	1,354 (45.1%)
During the usage of isotretinoin, you may experience joint or back pain (yes)	1,165 (38.8%)
Nosebleed is one of the side effects of isotretinoin (yes)	1,075 (35.8%)
There is no relation between isotretinoin and depression (no, there is)	972 (32.4%)
While using isotretinoin, you may experience dizziness (yes)	909 (30.3%)
Isotretinoin does not cause dyslipidemia (no, it causes)	748 (24.9%)
Isotretinoin may lead to teratogenicity (fetal defects) if it is taken by a male (no)	592 (19.7%)
Isotretinoin can cause many long-term adverse effects (no)	295 (9.8%)
Patients’ knowledge item	
Isotretinoin can be used without prescription (no)	2,279 (75.9%)
Isotretinoin should be given with plenty of water to avoid dryness (yes)	2,165 (72.1%)
No need to have laboratory monitoring during isotretinoin use (no, there is a need)	1,912 (63.7%)
In sexually active females, two forms of contraceptive are a must before starting isotretinoin therapy (yes)	1,799 (59.9%)
Women must stop taking isotretinoin by at least 1 month before getting pregnant (yes)	1,513 (50.4%)
Two methods of contraception are required before starting treatment with isotretinoin (sexually active females) (yes)	1,188 (39.6%)
Lactation is allowed during isotretinoin therapy (no)	1,128 (37.6%)
Blood donation is allowed during isotretinoin therapy (no)	944 (31.5%)
Isotretinoin is recommended to be given with fatty meals (yes)	841 (28.0%)
Isotretinoin can be used for more than 6 months without stop (yes)	824 (27.5%)
Isotretinoin is recommended to be used with sunblock (yes)	777 (25.9%)
Total knowledge score (mean ± SD)	10.4 ± 5.99
Level of knowledge	
Poor	1,589 (52.9%)
Moderate	1,174 (39.1%)
Good	238 (7.9%)

When measuring the differences in the score of knowledge in relation to the sociodemographic characteristics of participants (Table 4), it was observed that a higher knowledge score was more associated with younger age (Z = 7.241; p < 0.001), female gender (Z = 9.937; p < 0.001), Saudi nationality (Z = 4.316; p < 0.001), never been married (Z = 7.994; p < 0.001) and previous usage of isotretinoin (Z = 18.907; p < 0.001). In contrast, students (H = 69.260; p < 0.001) and those living in the eastern region (H = 37.036; p < 0.001) were more associated with a lower knowledge score.

**Table 4:** Differences in the score of knowledge in relation to the participants’ sociodemographic characteristics (n = 3,001). ^a^: P-value is calculated using the Mann-Whitney Z test; ^b^: P-value is calculated using the Kruskal-Wallis H test. **: Significant at p-values <0.05.

Factor	Knowledge score (24), mean ± SD	Z/H-test	P-value
Age group ^a^			
≤25 years	11.2 ± 5.54	7.241	<0.001 **
>25 years	9.44 ± 6.39		
Gender ^a^			
Male	8.31 ± 6.46	9.937	<0.001 **
Female	11.0 ± 5.70		
Nationality ^a^			
Saudi	10.5 ± 5.97	4.316	<0.001 **
Non-Saudi	8.74 ± 6.06		
Region ^b^			
Central region	11.2 ± 5.71	37.036	<0.001 **
Eastern region	9.48 ± 6.29		
Northern region	10.6 ± 5.78		
Western region	10.8 ± 5.83		
Southern region	11.0 ± 5.59		
Marital status ^a^			
Never been married	11.2 ± 5.66	7.994	<0.001 **
Been Married	9.23 ± 6.31		
Occupational status ^b^			
Unemployed	9.16 ± 6.20	69.260	<0.001 **
Student	11.1 ± 5.54		
Employed	11.1 ± 6.55		
Have you ever used isotretinoin in the past? ^a^			
Yes	13.5 ± 4.55	18.907	<0.001 **
No	9.02 ± 6.04		

## Discussion

Despite some serious and teratogenic adverse effects, isotretinoin is by far the most cost-effective drug used compared to other medications for the treatment of moderate-to-severe acne [21-24]. Due to the increasing trend of isotretinoin-related use in Saudi Arabia, adequate understanding of this medication and knowledge about its safe use has become essential. Hence, this study aimed to evaluate the KAP of the general population regarding isotretinoin use. The results of this study showed that there was unsatisfactory knowledge among our participants. The overall mean knowledge score was 10.4 (SD = 5.99) out of 24 points. Based on the given criteria, 52.9% were categorized as having poor knowledge, 39.1% as moderate knowledge, and only 7.9% were categorized as having good knowledge levels. Consistent with our findings, Alshaalan [24] found that 60% of female acne patients had a low level of knowledge. This corroborates with the study of Shajeri et al., stating that the general population’s knowledge was poor, with a mean knowledge score of 2.1 (SD = 2.2) out of 9 points [25]. On the contrary, a study conducted among the Al-Madinah population reported satisfactory knowledge about isotretinoin, with 70% having heard of isotretinoin, and over 60% knowing of its side effects, which were consistent with the study done in the Qassim region [26,27]. Better knowledge can be achieved by continuous health education, and awareness campaigns are necessary to educate the public about isotretinoin and its safe use.

Better isotretinoin knowledge was significantly associated with being younger, female gender, Saudi nationality, being single, and having previously used Isotretinoin. However, students living in the eastern region were significantly associated with lower knowledge scores. In Jeddah [28], a study documented an association between gender and knowledge about medication side effects along with safety precautions that pregnant women should be aware of during medication use, wherein females exhibited better knowledge and awareness of the necessary measures. This is comparable with the study of Jarab et al. [29], who reported female gender and isotretinoin use for more than six months to be significantly associated with a higher knowledge score. However, in the study by Molla et al. [26], the age group showed a significant relationship with knowledge about Roaccutane, future plans, and previous use of Roaccutane.

Assessing the specific details of isotretinoin side effects knowledge, the results of our study were conflicting. For instance, our respondents were aware of the association between isotretinoin use and skin dryness (75%), that it can lead to teratogenicity if taken during pregnancy (62%), and that increasing dose was associated with increasing side effects. However, most of our respondents had poor knowledge of other determinants of side effects, particularly about isotretinoin’s influence on dyslipidemia (24.9%), the no effect of teratogenicity on males (19.7%), and whether there are any long-term side effects of isotretinoin use (9.8%). Among the general public in Al-Ahsa [30] and acne patients in Riyadh [31], approximately 89% recognized teratogenicity as the most dangerous side effect associated with isotretinoin use; however, some of their respondents were not aware that they must cease taking isotretinoin at least six months before pregnancy. In Turkey [32], skin dryness was the most prominent side effect of isotretinoin, and while most patients accepted oral isotretinoin treatment (86.4%), few (14.4%) who were aware of its side effects refused to use the treatment.

Regarding the general isotretinoin knowledge items, most respondents knew that isotretinoin could not be used without a prescription (75.9%). They understood that isotretinoin users should drink sufficient water to avoid dryness (72.1%). Moreover, they were knowledgeable about the importance of laboratory monitoring of isotretinoin’s effect (63.7%) and that the two forms of contraceptives were mandatory among sexually active females before isotretinoin initiation (59.9%). Our respondents exhibited better knowledge about the above contexts; however, a lack of understanding was seen in the following contexts: isotretinoin is recommended along with fatty meals (28%), isotretinoin can be used continuously for more than six months (27.5%), and sunblock should be used along with isotretinoin (25.9%). Among the isotretinoin users in Jeddah [32], 93% reported that the drug was prescribed by a physician, wherein 20 mg was the most common dose prescribed, with more than six months as the most common duration. Patients who used it for more than six months experienced dryness and loss of hair, and few were wary about the psychological burden of isotretinoin. Among Turkish parents [33], nearly two-thirds (62.5%) raised concerns about oral isotretinoin treatment, with 34.6% having a lack of understanding of whether the treatment is appropriate for people under 18 years, with 52.9% thinking that the users might damage their liver.

It is important to note that the prevalence of our population who previously used isotretinoin was 30.6%. Among isotretinoin users, nearly all (97.5%) used it to treat acne, with mild acne being the most common type (39%), which is not in accordance with the recommended guidelines. While 89% received a prescription from a dermatologist, few reported (2.7%) using without prescriptions. A similar prevalence of isotretinoin usage has been reported in Saudi Arabia among the general population [26,30] and pharmacy students [28], wherein authors recommended the implementation of methods to increase the safe use of isotretinoin and devise more effective regulations to control non-prescribed isotretinoin dispensing in Saudi Arabia.

Limitations

This study was subjected to some limitations. First, there was an unequal gender distribution; thus, we cannot generalize the knowledge between males and females. Second, being cross-sectional it is prone to disadvantages, including cause-and-effect relationships and bias. Lastly, an online survey is prone to bias as some participants might not be truthful with their answers to questions.

## Conclusions

The knowledge of the general population in Saudi Arabia regarding isotretinoin was insufficient. However, better knowledge was more frequently seen among younger females with a previous history of isotretinoin use. This study also provided evidence of students’ lack of knowledge about isotretinoin and its side effects. There is a need to increase awareness about isotretinoin use among our population, particularly among students. Collective efforts by authorities are needed to improve awareness. The promotion of safe isotretinoin utilization is important, and more effective regulations are vital to limit the unnecessary prescribing of isotretinoin in Saudi Arabia.
